# What difference can a year make? Findings from a survey exploring student, alumni and supervisor experiences of an intercalated degree in emergency care

**DOI:** 10.1186/s12909-019-1579-x

**Published:** 2019-06-06

**Authors:** Blair Graham, Hadir Elbeltagi, Pam Nelmes, Annie Jenkin, Jason E Smith

**Affiliations:** 1 Emergency Department, Derriford Hospital, Plymouth, PL6 8DH England; 2 Plymouth University, Drake Circus, Plymouth, PL4 8AA England

**Keywords:** Emergency medicine, Urgent care, Medical education, Medical school, Intercalated degree

## Abstract

**Background:**

One third of UK medical students undertake an intercalated degree, typically in traditional academic disciplines. It is less usual for students to undertake intercalated degrees that are directly aligned to a clinical speciality with longitudinal placements.

This cross sectional survey aims to explore the self-reported experiences of students, alumni and supervisors associated with a clinically oriented intercalated degree in emergency care featuring a longitudinal placement in a hospital emergency department over a 9-month academic year. Themes for exploration include student clinical and academic development, effect on career choice, supervisor experience and the effect on host institutions.

**Methods:**

Current students, previous alumni, and clinical placement supervisors associated with a single intercalated degree programme in urgent and emergency care since 2005 were identified from records and using social media. Separate online surveys were then developed and distributed to current students/ previous alumni and consultant physician supervisors, between May and August 2016. Results are presented using basic descriptive statistics and selected free text comments.

**Results:**

Responses were obtained from 37 out of 46 contactable students, and 14 out of 24 supervisors (80 and 63%, respectively). Students self-reported increased confidence in across a range of clinical and procedural competencies. Supervisors rated student competence in clinical, inter-professional and academic writing skills to be commensurate with, or in many cases exceeding, the level expected of a final year medical student. Supervisors reported a range of benefits to their own professional and personal development from supervising students, which included improved teaching and mentoring skills, providing intellectual challenge, and helping with the completion of audits and service improvement projects.

**Conclusions:**

Students report the acquisition of a range of clinical, academic, and inter-professional skills following their intercalated BSc year. A positive experience was reported by supervisors, extending to host institutions. Students reported feeling more enthusiastic about emergency medicine careers on completion. However, as students embarking on this degree naturally bring pre-existing interest in the area, it is not possible to attribute causation to these associations. Further investigation is also required to determine the longer term effect of clinically oriented intercalated degrees on career choice.

**Electronic supplementary material:**

The online version of this article (10.1186/s12909-019-1579-x) contains supplementary material, which is available to authorized users.

## Background

**Fig. 1:**
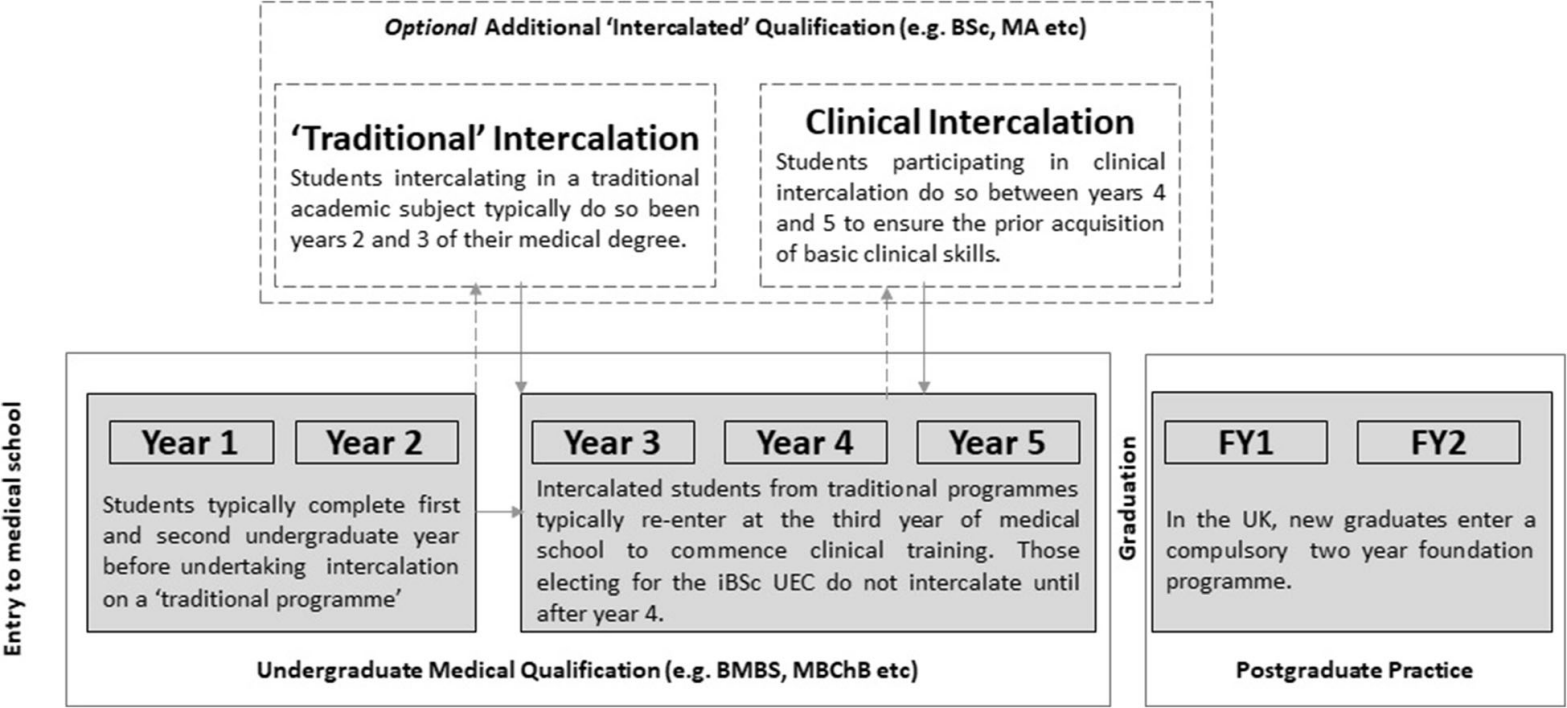
Schematic demonstrating progression of an undergraduate medical student in the UK. Students opting to undertake an intercalated degree typically do so between years two and three of study. In contrast, because the iBSc in emergency care focusses on clinical development, students are required to have completed their third and fourth years of study

Nationally, up to one third of UK medical students undertake an additional intercalated degree [1], typically in biomedical, social sciences, humanities, and arts related academic subjects. To do so, students usually interrupt their primary medical degree for one year, entering the final year of a non-medical degree programme which leads to the award of an additional bachelors’ or masters’ level qualification. Following intercalation, students return to complete their medical studies (Fig. 1). Reported benefits of intercalation include enhanced subsequent medical school performance, research skills, and an increased likelihood of selecting an academic career in the future [2, 3]. Whilst it remains unusual for students to have in depth clinical exposure as part of their intercalated degree, specific benefits of clinically oriented intercalated degrees have been explored in the context of primary care. When placed in this setting during an intercalated year, students reported enhanced development across a range of clinical, consultation and academic skills [4]. Nonetheless, exploration of a national UK database [5] revealed that out of 336 intercalated options available to UK students, only 22 programme titles were directly aligned with a clinical specialisation. Of these, only five programmes from three separate institutions explicitly offered a substantive clinical placement within their online prospectus. These were in emergency care (3 programmes) [6–8], critical care [9] and primary care [10]. As fewer than one in ten medical undergraduates intend to pursue clinical academic or research based careers [11], clinically based intercalated degrees may be more relevant for the majority.

The discrete benefits and challenges provided by a clinically focussed intercalated programme in urgent & emergency care have yet to be evaluated, though deficiencies in undergraduate acute care education have previously been recognised. Although relevant curricula and competency frameworks for acute care have been suggested [12, 13] none is currently mandated within UK medical schools. Indeed, a systematic review of 374 articles exploring undergraduate training in acute care concludes that undergraduates commonly ‘lack knowledge, confidence and competence in all aspects’. [14] Additional literature reports variable resuscitation training provision [15], low self-reported confidence amongst students across a range of emergency procedures [16] and difficulty consulting patients in the ED environment [17]. On the other hand, clinical placements in the ED have been demonstrated to offer more exposure to patient evaluation, decision making and procedures compared to other clinical placement settings and may be particularly helpful in overcoming some of these deficiencies [18]. Evaluating student experience of an urgent & emergency care intercalated degree that features a longitudinal clinical placement may yield insights into the effects on development, progression and career direction. In addition, the effect of hosting longitudinal placements from the perspective of supervisors and host institutions is currently unassessed.

This study aimed to explore the self-reported experiences of students and consultant supervisors towards a clinically oriented intercalated degree in urgent & emergency care. Themes identified for exploration included clinical and academic development, and effect on career choice, supervisor experience and host departments.

## Methods

### Design

Cross-sectional survey of enrolled students, alumni and supervisors associated with an intercalated Bachelor of Science degree in Urgent and Emergency care.

### Study setting

Plymouth University, situated in the South West of England, has offered medical students from across the UK an option to undertake the intercalated BSc in Urgent and Emergency Care (iBSc UEC) since 2005. Students undergo a competitive selection process during their fourth year of study. Those who are successful study alongside nurses, paramedics and allied health professionals to complete academic modules relating to the organisation of emergency care, leadership, quality improvement and paediatrics or mental health [19]. Although students complete summative assessments for each of these modules, they are not expected to produce a dissertation. Critical thinking, oral and written communication skills are instead evidenced through production of a professional portfolio and coursework including a quality improvement report, and are assessed alongside national criteria [20]. Crucially, each assessment is designed to reflect the complexity, autonomy and challenge required of a newly qualified medical graduate. For placement experience, students are assigned to one of the thirty emergency departments nationwide with which the Plymouth University has a formal working agreement, where they are expected to spend a minimum of thirty-seven hours per week in supervised placement, over a 9 month academic year. All students undergo health and criminal record screening, and are provided with an honorary contract with their host institution before being permitted to start placement. During induction, students are informed of their role, responsibilities and scope of practice in-keeping with regulatory guidance [21]. Students are supported throughout by an experienced emergency medicine consultant and the university programme team. A collaborative approach between the student, university and host institution is used to address any difficulties and concerns encountered, including the option to refer to a University fitness to practice panel, if necessary.

### Recruitment

Contact details of students, consultant supervisors and previous alumni who had been enrolled since 2005 were sought from existing records. Where this was not possible, the peer-to-peer messaging function of social networking software including Facebook, LinkedIn and Twitter were used by a single researcher (PN) to directly and confidentially contact potential participants. The value of using personal social media accounts, to extend a researchers ‘reach’ for the purpose of recruitment is increasingly recognised [22, 23]. Only details which already been made available in the public domain were accessed, and only a single attempt was made to contact each potential participant. Surveys were distributed to all respondents who agreed to participate by encrypted email with a further reminder sent two weeks later.

### Survey design

In order to assess the perspectives of students, alumni and supervisors, two separate surveys were required. Specific themes requiring exploration within each group were developed by consensus amongst the researchers. (Table 1).

**Table 1:** Selected themes for exploration, by survey

Student Survey		Supervisor Survey	
Theme under exploration (**bold** text) including brief descriptor	Item #	Theme under exploration (**bold** text) including brief descriptor	Item #
**Clinical Development** including technical, procedural and non- technical skills.	4–12	**Prior mentorship experience** with regards to undergraduate students	6–9
	13–15	**Prior teaching experience** with regards to undergraduate students	10–16
**Academic Development** including audit, research and quality improvement.	16–26	**Personal experience** of supervising iBsc students including time and workload commitments.	17–19
**General perceptions** of the iBSc UEC including student satisfaction.	27–32	**Institutional experience** of hosting iBSc students in the ED	20–24
Influence of the iBSc UEC on **career aspirations and future directions.**		**Subjective assessment of student performance** during the iBSc UEC including clinical and academic development	25–28

Themes were translated into survey items using categorical scales, and totalled 32 items for the student/alumni survey (Electronic Additional file 1), and 28 for supervisors (Electronic Additional file 2). To ensure relevance of items to real-world clinical practice, assessment of clinical competencies—including a predefined list of practical skills—was derived directly from existing UK Foundation Programme and emergency medicine curricula [24, 25]. Academic competencies were also derived from existing curricula where possible, and rating scales used to record perceptions of competence or skills acquisition were emulated from those featuring within Workplace Based Assessments used by UK medical trainees (Electronic Additional file 3). Otherwise, simple agreement scales were used. Space was also provided for open ended free text answers throughout.

The surveys were designed using an iterative process. The number of items was kept to a minimum to limit respondent fatigue and the survey was designed to be visually consistent and simple to follow. Once all researchers were satisfied with construction, independent external review was conducted by medical students and a consultant to provide an assessment of face validity and acceptability.

Ethical approval to conduct the study was granted by Plymouth University (reference 15/16–525).

### Survey administration

The survey was administered electronically using the *SurveyMonkey®* platform between May 2016 and August 2016.

The online platform allowed real time data collection which was transposed into Microsoft Excel for analysis.

## Results

Results are presented using descriptive statistics and selected free text comments.

A total of 95 students were enrolled on the programme between 2006 and 2016; contact details could be obtained for 46 of these (48.4% overall capture). Responses were ultimately obtained from 37 out of 46 contactable students and alumni (80%), the majority of whom were approaching graduation in 2016 (18/37), or were alumni who had graduated within the preceding two years (13/37). For the supervisor survey, 15 supervisors responded from 24 approached (63% overall capture). Completion rates were high for both surveys (86.4 and 93.3%, respectively). Open ended free text comments were also recorded.

### Student/alumni responses

Data relating to clinical and academic development, and the effect of the iBSc UEC on career aspirations and future directions are presented.

#### Clinical development

**Fig. 2:**
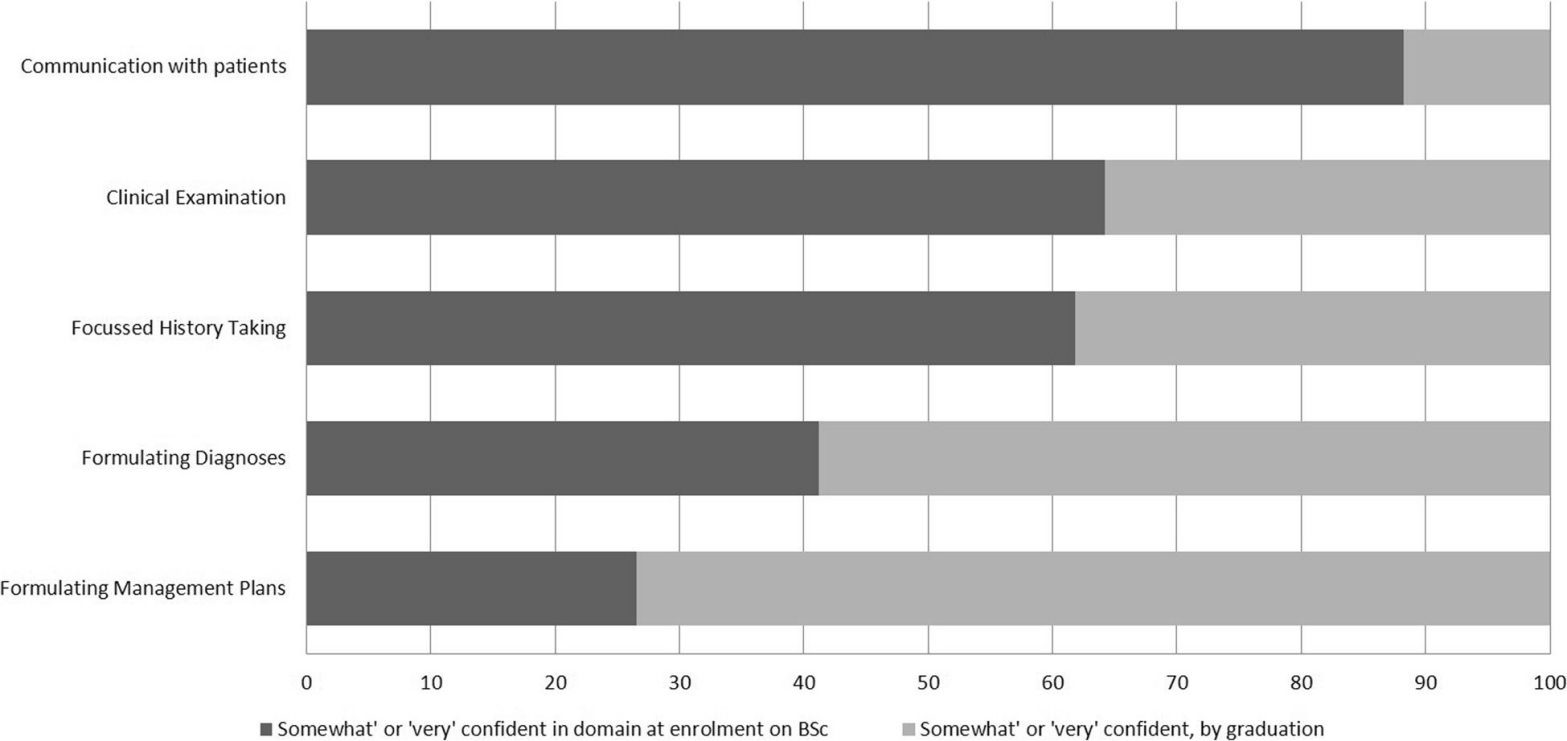
Bar Chart illustrating student/ alumni self-reported exposure to a range of generic clinical competencies prior to, and following the BSc, %

By the time of graduation from the iBSc UEC, all respondents reported that they felt either ‘somewhat’ or ‘very confident’ in performing a range of generic competencies including ‘communication with patients’, ‘focussed history taking’, ‘clinical examination’, and ‘formulating a diagnosis and management plan’ (Fig. 2). The largest increases in subjective confidence was observed in formulating a management plan’(100% at graduation vs. 26.5% on enrolment), formulating differential diagnoses (100% vs. 41.2%) and taking a history (100% vs. 61.8%).

**Fig. 3:**
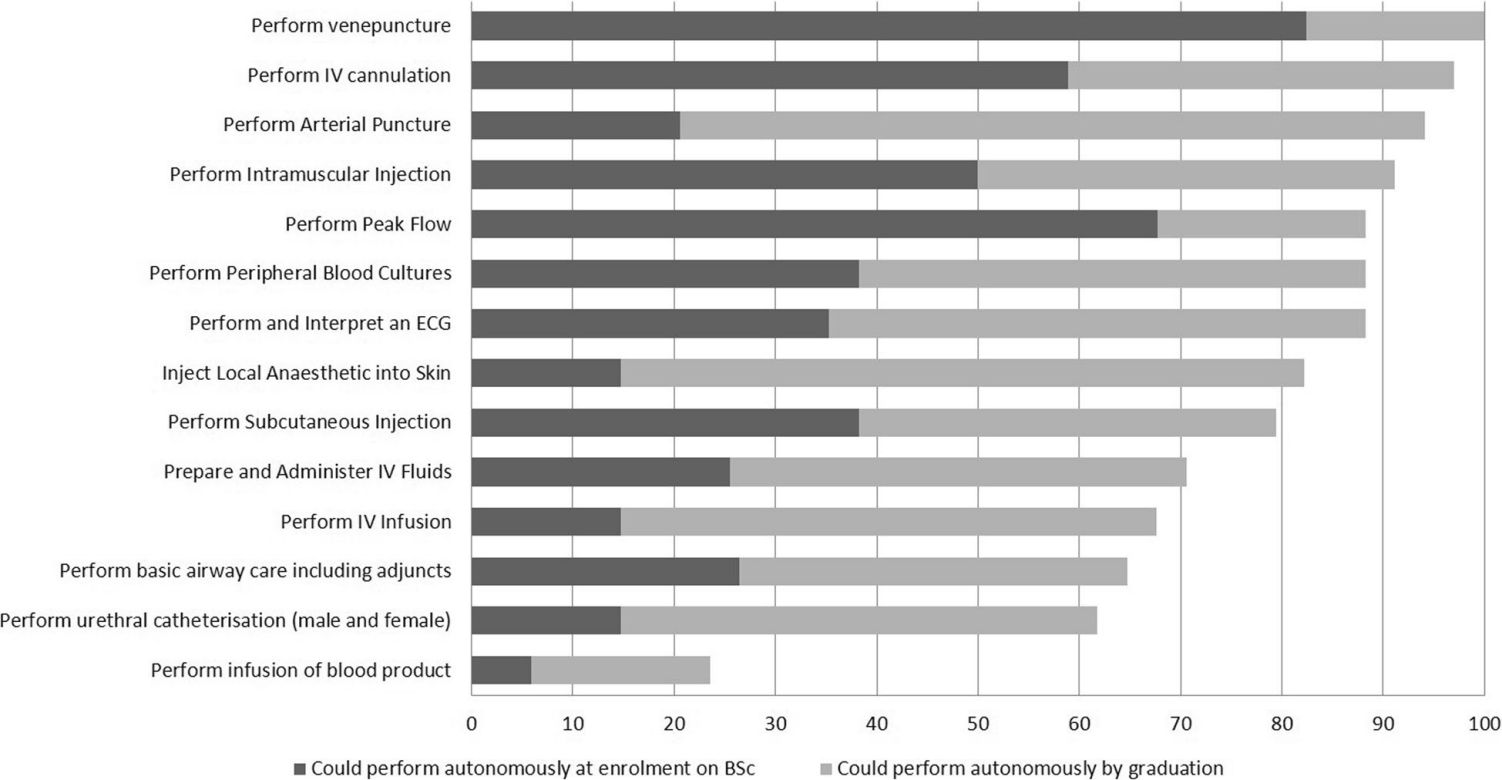
Bar Chart illustrating student/ alumni self-reported ability to autonomously perform clinical procedures before, and following the BSc, %

By the time of graduation, respondents reported being most confident with skills including venepuncture (100% on graduation vs. 82.5% on enrolment), cannulation (97% vs. 59%), arterial puncture (94% vs. 20.5%), intramuscular injection (91.2% vs. 50%), performing and analysing an ECG (88.2% vs. 35.2%) and peak flow measurement (88.2% vs. 67%) (Fig. 3).

Within free-text comments, respondents also mentioned the acquisition of skills in communication, handover, making patient referrals, teamwork, leadership, and teaching others, which they felt had been gained during the iBSc UEC year. These are summarised in Table 2.

**Table 2:** Summary of specific non-technical skills acquisition reported by respondents

***Are there any additional skills you feel that you have gained during the iBSc year?***	
“Handover, including SBAR” Respondent #30	
“Handover and referrals” Respondent #29	
“Leadership knowledge and skills” Respondent #23	
“Teamwork, teaching… knowledge of ED flow and how the ED works” Respondent #18	
“Team work” Respondent #6	

#### Academic development

Most respondents reported participation in clinical audit (26/32; 81.3%). Although a smaller number of respondents reported direct involvement in designing and conducting research (6/32; 18.8%) this translated into a high success rate at publication (5/6; 83%). Over half of respondents gained some experience presenting academic findings at scientific meetings (18/32; 56%). Several students received formal training in research, including meta-analysis and research governance (12/32; 37.5%). Additional participation in academic activities included developing and teaching simulation sessions, assisting with the delivery of life support courses, and undertaking quality improvement projects. Despite this, several students expressed a desire to be more involved with clinical research, indicating that they were frustrated with a perceived lack of opportunities which may have been due to operational pressures on departments and supervisors:

*“I would have liked more encouragement and help from my mentor to engage in research to present at conferences or meetings.”*

*Respondent # 36*

*“I would have liked to have written / helped to write a case-report or paper to publish. Department was incredibly pressured over the winter and I think everyone was too busy / stressed to consider additional academic exercises.”*

*Respondent #21*

#### Career aspirations and future directions

The effect of the iBSc UEC on future career development was also evaluated. All respondents agreed that the course encouraged skills development for future progression both as medical students and junior doctors. Similarly, all agreed that the programme increased their interest in a career in emergency medicine. A range of positive comments were obtained highlighting personal benefits of the course for future practice and consideration of emergency medicine as a future career:

*“I feel the BSc gave me a greater insight into the world of Emergency Medicine and as a result strengthened my interest in this speciality as a career choice.”*

*Respondent #26*

*“The best decision I made with regards to career progression. It helped to build my confidence which subsequently made starting F1 less daunting. My clinical skills improved immensely and I feel that this has made me a more competent junior doctor.”*

*Respondent #20*

Even where a previous student felt unable to consider pursuing a career in emergency medicine due to personal circumstances, she did not regret taking a year to do the iBSc UEC:

*“…My own personal circumstances have meant I am no longer considering a career in emergency medicine in the current climate …. Either way at the time taking the BSc was the right choice and I thoroughly enjoyed it.”*

*Respondent #31*

The single stated disadvantage to intercalation was the additional financial pressure incurred as a result of taking a year of extra study, reported by 11 students (31%).

### Consultant supervisor responses

#### Mentorship and teaching experience

The fifteen consultant respondents had been supervisors on the course for an average of 3 years (range 1–11 years). All reported that their mentorship role included goal and objective setting, holding regular meetings with students and providing pastoral and career advice. Supervisors reported spending an average of 2 h supervising students per week (range 0.5–3 h). All supervisors had previous experience of teaching at an undergraduate level.

#### Personal experience of supervising iBSc students

When asked about the effect of supervising iBSc UEC students on their wider attitude to undergraduate education, some consultants reported increased personal enthusiasm and engagement with undergraduate teaching as a direct result (6/15; 40%). Consultants reported a range of benefits to their own professional and personal development from supervising iBSc UEC students which included improved teaching and mentoring skills, providing additional intellectual challenge, and helping with the completion of audits and quality improvement projects:

*“I have found it to be very useful to have students in the department who question and challenge the status quo. Having keen intercalated students around means that they can assist with audit and research projects.”*

Supervisors also remarked that hosting the iBSc UEC students increased their personal career satisfaction:

*“[Hosting BSc students] Improves my training, mentoring and teaching skills. Helps to keep my enthusiasm for EM going by helping to inspire the student.”*

*Consultant #1*

*“Enthusiastic students make the job worthwhile and seeing them develop and succeed reminds me of the importance of passing on experience and supporting them in their careers.”*

*Consultant #6*

Consultant supervisors highlighted challenges relating to the time required to support an iBSc UEC student, the effect on medical students on a shorter placement and lack of personal remuneration.

*“There is no time allocated within job plans to supervise these students.”*

*Consultant #12*

*“Overload of students in the department which can have a negative effect.”*

*Consultant #2*

However, no supervisors reported clinical disadvantages to hosting a student. As a solution to these challenges, one consultant remarked that supervision was shared amongst colleagues within the department:

*The student’s supervision is done by colleagues right across the department and this has been a rewarding experience for all concerned. In addition it means the students are exposed to a range of different perspectives and practice styles.*

*Consultant #1*

#### Institutional effects of hosting iBSc students

In addition to personal benefits of mentoring a student, all consultant supervisors agreed that hosting a BSc UEC student had a positive effect on the wider ED. In particular, the perceived positive effect of a resident BSc UEC student on benefiting other students was noted by one:

*“The students become embedded in the department and therefore raise the profile of all students in a positive way”.*

*Consultant #14*

#### Subjective assessment of students’ performance

**Fig. 4:**
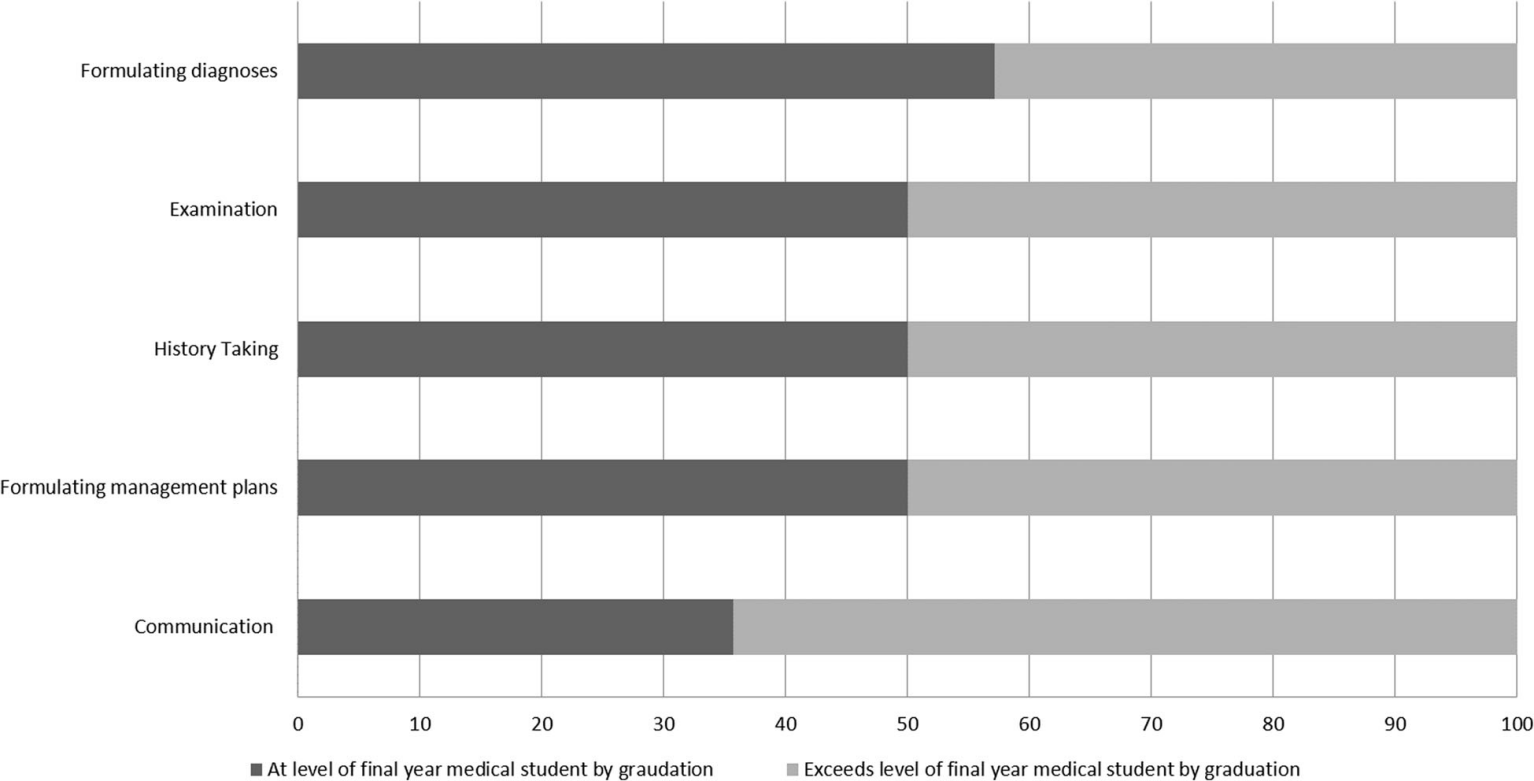
Bar Chart illustrating supervisor assessment of BSc student competence in clinical domains following BSc, %

Supervisors reported an assessment of their most recent student’s clinical and academic performance. All students performed at least to the level expected of a final year medical student by the end of the iBSc UEC year in these domains, with at least half exceeding expectations within each domain (i.e. at the level expected of a Foundation Year 1 (FY1) doctor) (Fig. 4).

**Fig. 5:**
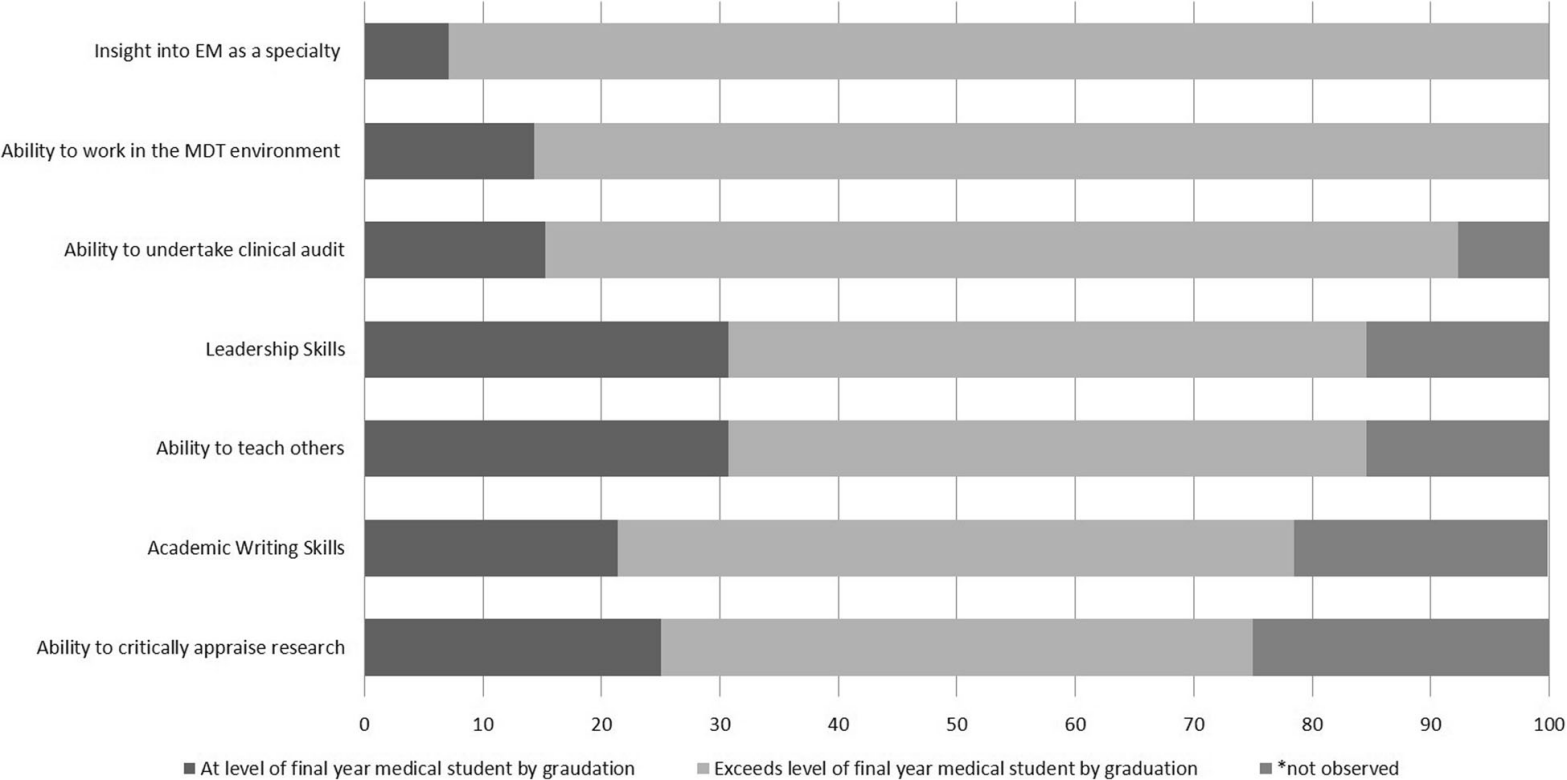
Bar Chart illustrating supervisor assessment of BSc student competence in non-clinical domains following the iBSc, %

Similarly, supervisors assessed acquisition of competence in other areas including teamwork, teaching, leadership, audit, and academic writing skills. Again—where observed—all students reached expectation for a final year medical student, and a large proportion exceeded this expectation and performed to the level of an FY1 or FY2 doctor—especially in the domains of insight into EM as a specialty (92.3% exceeding expectations), ability to work in the multidisciplinary team environment (85.7% exceeding expectations) and ability to undertake clinical audit (77% exceeding expectations) (Fig. 5).

## Discussion

**Fig. 6:**
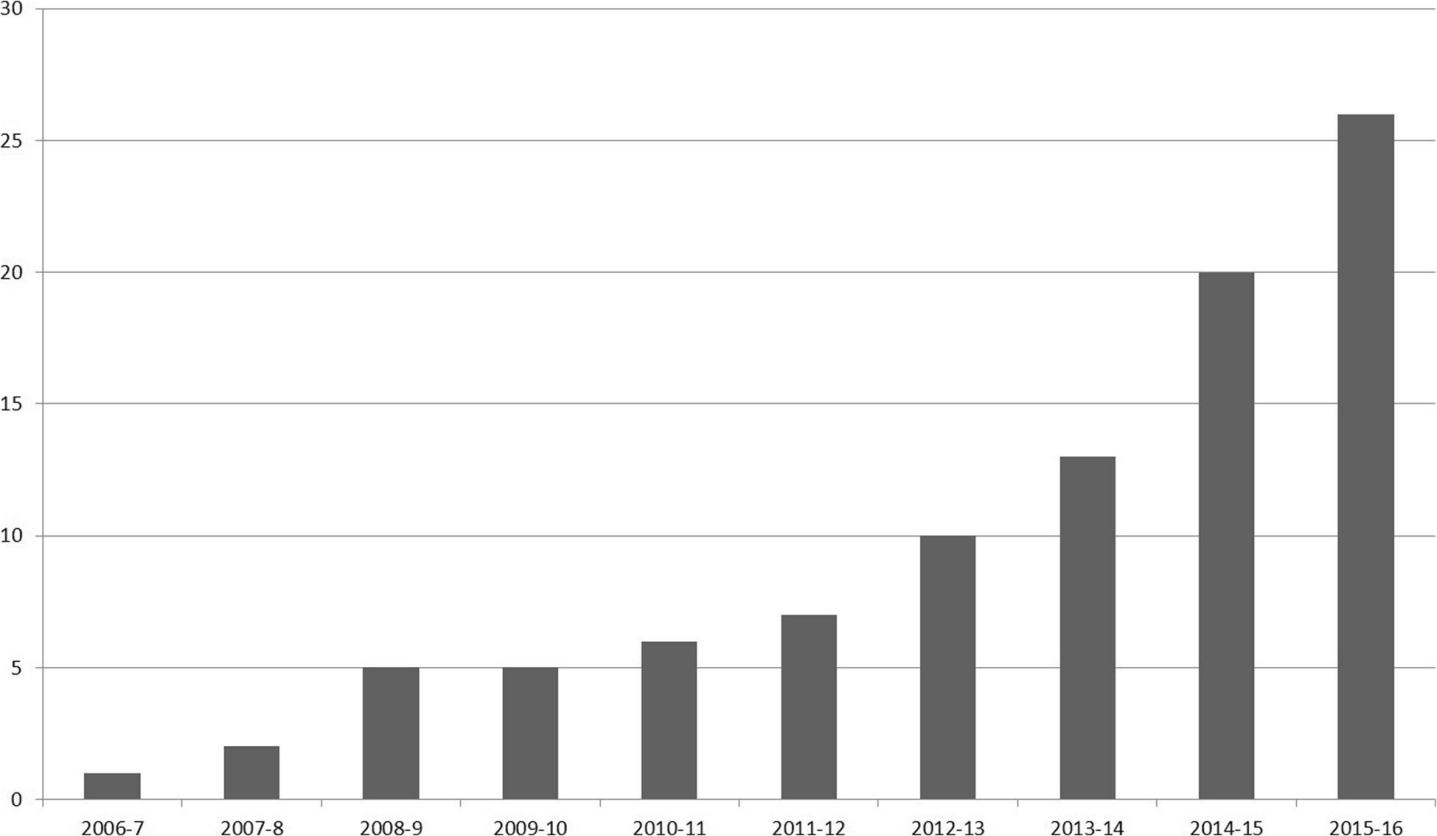
Bar chart demonstrating numbers of students enrolled on iBSc UEC during study period (2005—2016)

There is increasing emphasis on developing medical students’ clinical competence at an undergraduate level [24–27]. Whilst it largely remains the norm for intercalated medical students to study traditional academic subjects, many students cite lack of interest in traditional subjects, research and academic medicine, and as reasons for not wishing to pursue an intercalated degree [2, 28]. Programmes that feature supervised placements may offer a compelling alternative for those who wish to focus on clinical development, explore a specialty area in detail, and inform their future career choice. Interest in such programmes may be increasing—the BSc UEC alone has experienced an exponential increase in cohort size since its inception, which has increased from one student in 2005 to twenty-six students by 2016 (Fig. 6) and other institutions now offer such programmes.

Students reported developing confidence in a range of clinical and practical skills during the iBSc year. Supervisors universally reported that students typically met the expected competence thresholds for a final year undergraduate by the end of their intercalated year. In many cases, students exceeded this and were assessed as capable of performing at a level expected of a more experienced junior doctor. Indeed, all students and alumni felt that the course prepared them well for their subsequent final undergraduate year and practice as a foundation doctor. Evidence suggests that UK medical students may lack preparedness for practice as a foundation doctor [29–31] specifically in acute care [12–16, 30, 31], resuscitation [14], and also the emotional aspects of dealing with emergency situations [31]. Results from this study confirm that a longitudinal placement in emergency care may be particularly effective in addressing some important gaps. As many skills developed in the ED have generic relevance to practice elsewhere in the hospital and community, the positive impact of intercalation is likely to have applicability beyond a future placement or career choice in emergency medicine alone. The importance of non-technical skills acquisition during medical school is increasingly recognised [32], and it is encouraging that several respondents mentioned development of professional communication, leadership and team working skills during the iBSc UEC.

Most students reported active engagement with clinical audit and several undertook additional training in research methods and governance. This is in keeping with students’ experiences in other unrelated programmes [33]. Although not all students reported participation in clinical research, those who did had a high success rate at publication, with over half presenting findings of work at scientific meetings. Some students reported within the survey that they would have liked more opportunity to engage with research and academic activity within the ED. As a direct result of the findings of this survey, all students now receive seminars on academic emergency medicine and critical appraisal, and are supported by academic emergency physicians to develop their own research ideas.

Free-text answers yield insight into the development of a broader range of clinical, academic and management activities than were initially anticipated during the survey design phase. Although the concept of the ‘hidden curriculum’ is well documented within medical education [34], it is possible that students who embark on a longitudinal placement may gain especially detailed insights into ‘real world’ practice that otherwise might not occur until after graduation. As such, we suggest that an accelerated transition from ‘undergraduate student’ to ‘real world practitioner’ may exist for students embarked on a clinically oriented intercalation programme. Whilst such preparation may stand to positively enhance the transition from undergraduate to postgraduate roles, the potential for the development of moral distress and secondary traumatic stress is recognised in medical students working in acute settings [35, 36] and whether such phenomena are more likely during longitudinal placements warrants specific exploration. The additional financial burden posed by an additional year of study has also been recognised as a potential barrier to intercalation [37] although UK domestic students intercalating in their fifth year of study can currently expect to receive a National Health Service Bursary for tuition fee support [38].

### Limitations

Both surveys were externally peer reviewed prior to dissemination, but did not undergo formal validation due to the small numbers of participants involved. Nonetheless, the high completion rate for both indicates high acceptability. It was not possible to contact previous alumni in all cases. Whilst the potential for response bias exists, the high capture rate amongst contactable participants is reassuring. To limit the effects of social desirability bias, both surveys were fully anonymised. Most respondents had recent involvement with the programme within the prior three years, making significant recall bias less likely. Whilst it is encouraging that the BSc seems to engender enthusiasm towards careers in emergency medicine, further longitudinal follow up is required before any effect longer term career choices can be ascertained. Importantly, the study provides an assessment of clinical exposure for key conditions and the development of students’ confidence across a wide range of skills and competencies during the iBSc UEC year. Although student and alumni perceptions of confidence are self-reported, these are supported by consultant supervisor subjective assessments of competence. Finally, it is recognised that students choosing to intercalate in Urgent & Emergency care are by definition self- selecting and will have some pre-existing interest in this area. The acquisition of confidence and subsequent performance may have been derived from experiences in addition to those provided the intercalated programme. As such, it is not possible to attribute causation to the positive associations found in this study.

## Conclusions

Traditionally, the focus of intercalated degrees has been to develop students’ academic and research skills. Clinically oriented programmes may expand the remit of intercalated programmes by giving students an opportunity to embed themselves within a host clinical department over an entire academic year and acquire a broad range of clinical competencies and skills. The majority of students who responded to this study also reported engagement in academic activities including clinical audit and research. Therefore, clinically focussed intercalated degrees may have the potential to provide students with the ‘the best of all worlds’. Offering undergraduates in-depth exposure to a clinical area of interest during medical school may help inform career choice, perhaps standing to improve the recruitment and retention of trainees in the longer term. Institutions wishing to increase undergraduate participation in certain clinical specialties may wish to consider the potential merit of initiating similar programmes.

We are currently planning a longitudinal study to build on the findings presented here and specifically further understand how the iBSc influences development and career choice of graduates.

## Additional files

Additional file 1: Student/ Alumni survey. (PDF 636 kb)

Additional file 2: Consultant supervisor survey. (PDF 325 kb)

Additional file 3: Use of scales in the surveys. Students and alumni were requested to record perceptions of competence across a range of defined clinical skills *before* and *after* the BSc using a five- point scale that is based on that used by ACCS trainees. b Students rate general perceptions of the course using a standard five point agreement scale. c Supervisors were requested to rate student competence using a scale to determine equivalence or superiority with various stages of training. This was extended to FY2 level, to reflect. Anecdotal reports of some students operating to this level in certain domains by the end of their BSc and ED placement. (DOCX 315 kb)

## Abbreviations

ED: Emergency Department
FY1: Foundation Year 1 Doctor
FY2: Foundation Year 2 Doctor
GCP: Good Clinical Practice
iBSc UEC: Intercalated Bachelor of Science Degree in Urgent and Emergency Care
