# Supplementary material for: What difference can a year make? Findings from a survey exploring student, alumni and supervisor experiences of an intercalated degree in emergency care
Source: BMC Med Educ. 2019 Jun 6;19:188. doi: 10.1186/s12909-019-1579-x (PMC6554867; doi:10.1186/s12909-019-1579-x)
Supplement: Supplementary file 1 — Student/ Alumni survey. (PDF 636 kb) [file 12909_2019_1579_MOESM1_ESM.pdf]

Participant information

Final call for responses!
***Please* complete before 30/06/2016**

Thank you for considering taking part in this survey, to evaluate your experiences of the BSc in emergency care programme. Below is some information regarding the survey. Please read this before commencing the survey.

Why have I been selected?
You have been identified as a current or previous intercalated BSc student enrolled on the emergency care programme at Plymouth University.

What are the aims of the survey?
The survey aims to evaluate your experiences of the BSc programme in the following domains:
- **Clinical Skills**
- **Academic Leadership and Management Skills**
- **Career preparation and progression**

The survey will also ask you to rate your general perceptions of the BSc programme.

How will the results be used?
The results will be used by the existing BSc faculty to evaluate the effectiveness of the current programme. Results may be used to improve the relevance of the programme content and delivery for future intercalated students.

The results may also be published in peer reviewed journals and / or presented at educational meetings.

Will the results be anonymised?
Yes. This survey does not collect any of your personal identifiable information.
Do I have to take part?
No. You participation is entirely voluntary.

How long do I have to take part?
The initial survey window is from 06/05/2016 to 13/06/2016. Please complete the survey within this time if you wish to take part.
How long will completing the survey take?
The online survey should take about 15-20 minutes to complete

Can I get a copy of the final report?
Yes- please email blair.graham1@nhs.net to request a copy of the final report when it is published.
I have further questions. Who can I contact?
Please email Dr Blair Graham blair.graham1@nhs.net or Pam Nelmes

(p.nelmes@plymouth.ac.uk) to request further information.

Stage 1 out of 7: About You

Estimated time to complete: 2 minutes

1. What is your gender?

- ○ Female
- ○ Male

2. Which race/ethnicity best describes you? (Please choose only one.)

- ○ White
- ○ Mixed/ Multiple ethnic groups
- ○ Black/ African/ Caribbean/ Black British
- ○ Other ethnic group
- ○ Would rather not say

3. What year did you commence the BSc?

[ ]

Stage 2 of 7 : Clinical Skills

Estimated time to complete: 10 minutes

Questions 4-12 explore your clinical skills development prior to and following the intercalated BSc

4. 1. How confident were you in the following domains prior to starting the BSc?

	Not confident at all	Somewhat unconfident	Somewhat confident	Very confident
a. Communication with patients	◯	◯	◯	◯
b. Focussed History Taking	◯	◯	◯	◯
c. Clinical Examination	◯	◯	◯	◯
d. Diagnosis	◯	◯	◯	◯
e. Formulating a management plan	◯	◯	◯	◯

5. 1. How confident were you in the following domains after completing the BSc course?

	Not confident at all	Somewhat unconfident	Somewhat confident	Very confident
a. Communication with patients	◯	◯	◯	◯
b. Focussed History Taking	◯	◯	◯	◯
c. Clinical Examination	◯	◯	◯	◯
d. Diagnosis	◯	◯	◯	◯
e. Formulating a management plan	◯	◯	◯	◯

6. Before starting the course, did you have any experience of assessing and managing patients with the following conditions

	No experience	Background/ theoretical training but **no** actual cases observed	Observed a single case in practice	Observed multiple cases in practice
a. Anaphylaxis	◯	◯	◯	◯

If you 'observed multiple cases in practice', how many did you observe and during which medical school placement(s)?

	No experience	Background/ theoretical training but **no** actual cases observed	Observed a single case in practice	Observed multiple cases in practice
b. Cardiorespiratory Arrest	○	○	○	○

If you 'observed multiple cases in practice', how many did you observe and during which medical school placement(s)?

[ ]

	No experience	Background/ theoretical training but **no** actual cases observed	Observed a single case in practice	Observed multiple cases in practice
c. Major Trauma	○	○	○	○

If you 'observed multiple cases in practice', how many did you observe and during which medical school placement(s)?

[ ]

	No experience	Background/ theoretical training but **no** actual cases observed	Observed a single case in practice	Observed multiple cases in practice
d. Septic Patient	○	○	○	○

If you 'observed multiple cases in practice', how many did you observe and during which medical school placement(s)?

[ ]

	No experience	Background/ theoretical training but **no** actual cases observed	Observed a single case in practice	Observed multiple cases in practice
e. Shocked Patient	○	○	○	○

If you 'observed multiple cases in practice', how many did you observe and during which medical school placement(s)?

[ ]

	No experience	Background/ theoretical training but **no** actual cases observed	Observed a single case in practice	Observed multiple cases in practice
f. Unconscious Patient	○	○	○	○

If you 'observed multiple cases in practice', how many did you observe and during which medical school placement(s)?

[ ]

	No experience	Background/ theoretical training but **no** actual cases observed	Observed a single case in practice	Observed multiple cases in practice
g. Minor Musculoskeletal Trauma	○	○	○	○

If you 'observed multiple cases in practice', how many did you observe and during which medical school placement(s)?

[ ]

	No experience	Background/ theoretical training but **no** actual cases observed	Observed a single case in practice	Observed multiple cases in practice
h. Wound Care	○	○	○	○

If you 'observed multiple cases in practice', how many did you observe and during which medical school placement(s)?

[ ]

	No experience	Background/ theoretical training but **no** actual cases observed	Observed a single case in practice	Observed multiple cases in practice
i. Febrile Child	○	○	○	○

If you 'observed multiple cases in practice', how many did you observe and during which medical school placement(s)?

[ ]

	No experience	Background/ theoretical training but **no** actual cases observed	Observed a single case in practice	Observed multiple cases in practice
j. Acute Psychiatric Illness	○	○	○	○

	No experience	Background/ theoretical training but **no** actual cases observed	Observed a single case in practice	Observed multiple cases in practice

If you 'observed multiple cases in practice', how many did you observe and during which medical school placement(s)?

7. During the BSc, did you gain experience of assessing and managing patients with the following conditions? (tick all that apply)

	No additional experience gained during BSc	Gained additional background / theoretical training during BSc	Observed a single case in practice during the BSc	Observed multiple cases in practice during the BSc
a. Anaphylaxis	☐	☐	☐	☐
b. Cardiorespiratory Arrest	☐	☐	☐	☐
c. Major Trauma	☐	☐	☐	☐
d. Septic Patient	☐	☐	☐	☐
e. Shocked Patient	☐	☐	☐	☐
f. Unconscious Patient	☐	☐	☐	☐
g. Minor Musculoskeletal Trauma	☐	☐	☐	☐
h. Wound Care	☐	☐	☐	☐
i. Febrile Child	☐	☐	☐	☐
j. Acute Psychiatric Illness	☐	☐	☐	☐

8. At the beginning of the BSc, what was your level of experience in the following practical skills…

	No experience- could not perform	Always needed direct supervision/ help to perform	Sometimes needed direct supervision/ help to perform	Could perform autonomously but not formally assessed	Could perform autonomously and formally assessed
Venepuncture	○	○	○	○	○
IV Cannulation	○	○	○	○	○
Prepare and administer IV medication and injections and fluids	○	○	○	○	○
Arterial puncture in an adult	○	○	○	○	○
Blood culture (peripheral)	○	○	○	○	○
IV infusion including the prescription of fluids	○	○	○	○	○
IV infusion of blood and blood products	○	○	○	○	○
Injection of local anaesthetic to skin	○	○	○	○	○
Subcutaneous injection	○	○	○	○	○
Intramuscular injection	○	○	○	○	○
Perform and interpret an ECG	○	○	○	○	○
Perform and interpret peak flow	○	○	○	○	○
Urethral catheterisation (male and female)	○	○	○	○	○
Airway care including simple adjuncts	○	○	○	○	○

Were there any additional clinical skills or knowledge areas you developed which are not covered above?

9. By the end of the BSc Course, what was your level of experience in the following practical skills…

	No experience- could not perform	Always needed direct supervision/ help to perform	Sometimes needed direct supervision/ help to perform	Could perform autonomously but not formally assessed	Could perform autonomously and formally assessed
Venepuncture	○	○	○	○	○
IV Cannulation	○	○	○	○	○
Prepare and administer IV medication and injections and fluids	○	○	○	○	○
Arterial puncture in an adult	○	○	○	○	○
Blood culture (peripheral)	○	○	○	○	○
IV infusion including the prescription of fluids	○	○	○	○	○
IV infusion of blood and blood products	○	○	○	○	○
Injection of local anaesthetic to skin	○	○	○	○	○
Subcutaneous injection	○	○	○	○	○
Intramuscular injection	○	○	○	○	○
Perform and interpret an ECG	○	○	○	○	○
Perform and interpret peak flow	○	○	○	○	○
Urethral catheterisation (male and female)	○	○	○	○	○
Airway care including simple adjuncts	○	○	○	○	○

10. Were there any additional skills you developed during the course of the BSc, not mentioned above?

11. Were there any additional skills you would have liked to develop during the course of the BSc, but were not able to?

12. If you gave an answer to Q11, what were the barrier(s) to developing these skills?

Stage 3 of 7: Academic and Leadership Skills

Estimated time to complete: **5 minutes**

Questions 13-15 explore the development of academic and leadership/management skills during the BSc.

13. During the BSc were you able to?

	No	Yes
a. Write a research proposal?	○	○
If 'yes' please give relevant further details (e.g. title of work, conference / management activity attended) [ ]		
b. Gain any experience of the ethics approval process?	○	○
If 'yes' please give relevant further details (e.g. title of work, conference / management activity attended) [ ]		
c. Gain any experience of applying for research funding?	○	○
If 'yes' please give relevant further details (e.g. title of work, conference / management activity attended) [ ]		
d. Gain a good clinical practice certificate?	○	○
If 'yes' please give relevant further details (e.g. title of work, conference / management activity attended) [ ]		
e. Gain specific training in any methods relevant to research?	○	○
If 'yes' please give relevant further details (e.g. title of work, conference / management activity attended) [ ]		
f. Author or co-author a peer reviewed publication?	○	○
If 'yes' please give relevant further details (e.g. title of work, conference / management activity attended) [ ]		
g. Present your work (poster/oral presentation) at a local or regional conference?	○	○

	No	Yes
If 'yes' please give relevant further details (e.g. title of work, conference / management activity attended)		
h. Present your work (poster/oral presentation) at a national conference?	◯	◯
If 'yes' please give relevant further details (e.g. title of work, conference / management activity attended)		
i. Present your work (poster/oral presentation) abroad at an international conference?	◯	◯
If 'yes' please give relevant further details (e.g. title of work, conference / management activity attended)		
j. Receive any funding to attend a conference or other research related activities?	◯	◯
If 'yes' please give relevant further details (e.g. title of work, conference / management activity attended)		
k. Participate in a clinical audit?	◯	◯
If 'yes' please give relevant further details (e.g. title of work, conference / management activity attended)		
l. Complete a full audit cycle, including re-audit?	◯	◯
If 'yes' please give relevant further details (e.g. title of work, conference / management activity attended)		
m. Participate in other management/ leadership activity	◯	◯
If 'yes' please give relevant further details (e.g. title of work, conference / management activity attended)		

14. Were there any academic, leadership, or management opportunities or experiences you participated in not outlined above? (include as much detail as you can)

15. Were there any academic, leadership or management opportunities or experiences you wish you could have participated in? (include as much detail as you can)

Stage 4 of 7: Your perceptions of the BSc programme

Estimated time to complete 5 minutes

Questions 16-18 explore your general perceptions of the intercalated BSc

16. During the BSc programme, my…

	Strongly disagree	Generally disagree	Neither agree/ disagree	Generally agree	Strongly agree
a) teaching skills improved	◯	◯	◯	◯	◯
b) leadership skills improved	◯	◯	◯	◯	◯
c) academic writing skills improved	◯	◯	◯	◯	◯
d) understanding of the audit process improved	◯	◯	◯	◯	◯
e) understanding of the research process improved	◯	◯	◯	◯	◯
f) understanding of the management skills improved	◯	◯	◯	◯	◯
g) Interest in a career in emergency medicine increased.	◯	◯	◯	◯	◯
h) Ability at practical procedures improved	◯	◯	◯	◯	◯
i) Position within the ED seemed valued by wider team members	◯	◯	◯	◯	◯
j) Role was within the wider ED team was well defined	◯	◯	◯	◯	◯

17. During the BSc programme, I…

	Strongly disagree	Generally disagree	Neither agree/ disagree	Generally agree	Strongly agree
k) Became less satisfied with emergency care as a degree subject	◯	◯	◯	◯	◯
l) Developed skills useful for future progression in medical school	◯	◯	◯	◯	◯
m) Developed skills useful for future progression as junior doctors	◯	◯	◯	◯	◯
n) Incurred financial difficulty as a result of taking an additional year to intercalate	◯	◯	◯	◯	◯
o) Felt that the BSc represented poor value for money	◯	◯	◯	◯	◯

18. In summary, I would:

	Strongly disagree	Generally disagree	Neither agree/ disagree	Generally agree	Strongly agree
a. recommend the BSc to any future medical students	◯	◯	◯	◯	◯
c. have done a different intercalated degree in hindsight	◯	◯	◯	◯	◯

What other intercated degree(s) subjects may you have considered?

19. Have you graduated from the BSc programmme yet?

- ◯ Yes
- ◯ No

Stage 5 of 7: Influence career planning (undergraduate)

Estimated time to complete: 2 minutes

Questions 20-25 explore your undergraduate career progression following the BSc

20. Before the BSc , what three specialties were you most interested in applying for?

First prereference

Second Preference

Third Preference

21. Following the BSc , what three specialties were you most interested in applying for?

First prereference

Second Preference

Third Preference

22. If you have undertaken a medical elective since the BSc, where did you go? (please include country and institution)

23. If you have undertaken a medical elective since the BSc, what specilaty (or specialities) did you join?

24. Did the BSc help prepare you for your elective?

- No
- Yes
- Unsure/ Not Applicable

25. If you answered 'yes' to Q16, please give further details

26. Have you graduated from medical school?

- ◯ Yes
- ◯ No

Stage 6 of 7: Career planning and progression (postgraduate)

If you have not yet graduated from medicine and started work as a doctor, go to Stage 7.
Estimated time to complete 2 minutes

Questions 27-31 explore your undergraduate career progression following graduation as a doctor

27. Where do you work currently (institution)?

28. What is your current grade/ job title?

29. What is your current specialty?

30. Did the BSc help you prepare for practice as an FY1 doctor?

- Strongly Disagree
- Generally Disagree
- Neither agree/ disagree
- Generally Agree
- Strongly Agree

31. Have you noticed any other career benefits as a consequence of undertaking the BSc?

Stage 7 of 7: Additional Comments

32. Your general views relating to the BSc are also appreciated. Do you have any further comments to make?
