# Supplementary material for: What difference can a year make? Findings from a survey exploring student, alumni and supervisor experiences of an intercalated degree in emergency care
Source: BMC Med Educ. 2019 Jun 6;19:188. doi: 10.1186/s12909-019-1579-x (PMC6554867; doi:10.1186/s12909-019-1579-x)
Supplement: Supplementary file 2 — Consultant supervisor survey. (PDF 325 kb) [file 12909_2019_1579_MOESM2_ESM.pdf]

2016 BSc Urgent & Emergency Care Evaluation
Supervisor & Mentor Survey

Information for participants

Final call for responses!
***Please* complete before 30/06/2016**

Thank you for considering taking part in this survey, to evaluate your experiences of the BSc in Urgent and Emergency Care. Below is some information regarding the survey. *Please read this before commencing the survey.*

Why have I been selected?
You have been identified as a current or previous intercalated BSc supervisor for the emergency care programme at Plymouth University.

What are the aims of the survey?
The survey aims to evaluate your experiences of the BSc programme in the following domains:
· Mentorship
- Teaching
- Personal Workload
- Effects on wider department
- Assessment of benefits for students
The survey will also ask you to rate your general perceptions of the BSc programme.

How will the results be used?
The results will be used by the existing BSc faculty to evaluate the effectiveness of the current programme. Results may be used to improve the relevance of the programme content and delivery for future intercalated students.

The results may also be published in peer reviewed journals and / or presented at educational meetings.

Will the results be anonymised?
Yes. This survey does not collect any of your personal identifiable information, although we will ask which institution you are based in as this may be important when looking at overall results.

Do I have to take part?
No. Your participation is entirely voluntary.

How long do I have to take part?
The current survey window is from 06/05/2016 to 06/06/2016. Please complete the survey within this time if you wish to take part.

How long will completing the survey take?
The online survey should take about 15-20 minutes to complete

Can I get a copy of the final report?
Yes- please email blair.graham1@nhs.net to request a copy of the final report when it is

published.

I have further questions. Who can I contact?
Please email Dr Blair Graham (blair.graham1@nhs.net) or Pam Nelmes (p.nelmes@plymouth.ac.uk) to request further information.

Stage 1: About yourself

Estimated Time to complete: 2 minutes

1. What is your gender?

- ◯ Female
- ◯ Male

2. Which race/ethnicity best describes you? (Please choose only one.)

- ◯ White British
- ◯ Mixed/ Multiple Ethnicity
- ◯ Asian/ Asian British
- ◯ Black/ African/ Caribbean/ Black British
- ◯ Other
- ◯ Would rather not say

3. What is your current job title?

4. Where do you work?

5. How many years have you been supervising the BSc for?

Stage 2: Your development as a mentor

Estimated time to complete: 5 minutes

6. 1. What experience of mentorship did you have prior to supervising BSc students?

- ○ None at all
- ○ Informal (e.g. providing ad hoc leadership/ pastoral support to students/ juniors on shop floor)
- ○ Formal (e.g. educational/ clinical supervisor for students or postgraduate trainees)

Please state any formal roles:

[ ]

7. What mentorship roles do you undertake as a BSc supervisor? (tick all that apply)

- ☐ a. Specific goal/ objective setting
- ☐ b. Regular face to face meetings to discuss progress
- ☐ c. Pastoral care and advice
- ☐ d. Career guidance and advice
- ☐ e. All of the above

Other (please specify)

[ ]

8. Have you become more engaged in your role as a mentor since taking on your role as a BSc supervisor?

- ○ Definitley disagree
- ○ Disagree
- ○ Neither agree nor disagree
- ○ Agree
- ○ Strongly agree

9. Has your enthusiasm for acting as a mentor increased since taking on your role as a BSc Supervisor?

- ◯ Definitley disagree
- ◯ Disagree
- ◯ Neither agree nor disagree
- ◯ Agree
- ◯ Strongly agree

Section 3: Your development as a teacher

Estimated time to complete: 5 minutes

10. Do you have any formal teaching qualifications (e.g. PGCE)

- ○ Yes
- ○ No

If 'yes', please state qualification(s) (e.g. PGCE):

[ ]

11. 1. What experience of undergraduate teaching did you have prior to supervising BSc students?

- ○ a. None at all
- ○ b. Informal teaching role (e.g. shop floor teaching to students on placement, but no formal contract/ position within medical school)
- ○ c. Formal teaching role (e.g. any formal contract/ position in the medical school relating to undergraduate education)

Please state any formal roles:

[ ]

12. What teaching roles do you undertake as a BSc supervisor? (tick all that apply)

- ☐ a. Shop floor teaching
- ☐ b. Clinical tutorials
- ☐ c. Assessment of skills (e.g. practical procedures, examination)
- ☐ d. Feedback for written assessments
- ☐ e. Teaching as part of taught BSc modules (e.g. lectures)
- ☐ f. All of the above

Other (please specify)

[ ]

13. Have you become more engaged in your role as an undergraduate teacher since taking on your role as a BSc supervisor?

- ○ Definitley disagree
- ○ Disagree
- ○ Neither agree nor disagree
- ○ Agree
- ○ Strongly agree

14. Has your enthusiasm for teaching undergraduates changed since taking on your role as a BSc Supervisor?

- ◯ Definitley disagree
- ◯ Disagree
- ◯ Neither agree nor disagree
- ◯ Agree
- ◯ Strongly agree

15. Have you become more engaged in wider ED teaching since taking on your role as a BSc supervisor?

- ◯ Yes
- ◯ No
- ◯ Don't know/ NA

If you answered 'yes', please give example(s):

16. Has your enthusiasm for as a teacher increased since taking on your role as a BSc Supervisor?

- ◯ Definitley disagree
- ◯ Disagree
- ◯ Neither agree nor disagree
- ◯ Agree
- ◯ Strongly agree

Section 4: Personal time and workload

Estimated time to complete: 5 minutes

17. On average, how many hours do you dedicate to student supervision **per week**

18. What advantages, if any, does hosting a BSc student have on your personal and professional development?

19. What disadvantages, if any, does hosting a BSc student have on your personal and professional development?

Stage 5: Effects of hosting BSc students in your ED.

Estimated time to complete: 5 minutes

20. Hosting a BSc student has a positive overall effect on the ED

- Strongly disagree
- Disagree
- Neither agree nor disagree
- Agree
- Strongly agree

Additional comments:

21. The BSc student's role is well defined and understood by the wider team

- Strongly disagree
- Disagree
- Neither agree nor disagree
- Agree
- Strongly agree

Additional comments:

22. The BSc student's scope of practice is appropriate for their level of training (i.e. 4th/5th year medical undergraduate)

- Strongly disagree
- Disagree
- Neither agree nor disagree
- Agree
- Strongly agree

Additional comments:

23. Hosting a BSc student has presented some positive opportunities for me or my department

- ◯ Strongly disagree
- ◯ Disagree
- ◯ Neither agree nor disagree
- ◯ Agree
- ◯ Strongly agree

Please state any specific strengths below:

24. Hosting a BSc student has presented some disadvantages or risks for me or my department

- ◯ Strongly disagree
- ◯ Disagree
- ◯ Neither agree nor disagree
- ◯ Agree
- ◯ Strongly agree

Please state any specific disadvantages below:

Part 6: Assessment of benefits for students

Estimated time to complete: 5 minutes

25. By the end of the programme, I would rate my most recent student's ability in the following domains

	Below expectations for final year medical student	Meets expectations for final year medical student	Exceeds expectations for final year medical student	Meets expectations for FY1 doctor	Meets expectations for FY2 doctor	Exceeds expectations for FY2 doctor	Not observed/ cannot comment
Practical Skills	○	○	○	○	○	○	○
Clinical History	○	○	○	○	○	○	○
Clinical Examination	○	○	○	○	○	○	○
Formulating a differential diagnosis	○	○	○	○	○	○	○
Recommending a management plan for major conditions presenting to the ED	○	○	○	○	○	○	○
Teaching ability	○	○	○	○	○	○	○
Leadership skills	○	○	○	○	○	○	○
Academic writing skills	○	○	○	○	○	○	○
Ability to undertake an audit	○	○	○	○	○	○	○
Critical appraisal skills	○	○	○	○	○	○	○
Insight into emergency medicine	○	○	○	○	○	○	○
Ability to work within the wider ED team	○	○	○	○	○	○	○
Communication including handover and referrals	○	○	○	○	○	○	○

26. Are there any other areas of practice, not mentioned above, that you feel BSc students develop during the year?

27. In your opinion, are there any other positive effects of the BSc for students?

28. In your opinion, are there any other negative effects of the BSc for students?
