# Supplementary material for: What difference can a year make? Findings from a survey exploring student, alumni and supervisor experiences of an intercalated degree in emergency care
Source: BMC Med Educ. 2019 Jun 6;19:188. doi: 10.1186/s12909-019-1579-x (PMC6554867; doi:10.1186/s12909-019-1579-x)
Supplement: Supplementary file 3 — Use of scales in the surveys. Students and alumni were requested to record perceptions of competence across a range of defined clinical skills before and after the BSc using a five- point scale that is based on that used by ACCS trainees. b Students rate general perceptions of the course using a standard five point agreement scale. c Supervisors were requested to rate student competence using a scale to determine equivalence or superiority with various stages of training. This was extended to FY2 level, to reflect. Anecdotal reports of some students operating to this level in certain domains by the end of their BSc and ED placement. (DOCX 315 kb) [file 12909_2019_1579_MOESM3_ESM.docx]

**Electronic Supplementary Material 1**

| **(A)** Students and alumni were requested to record perceptions of competence across a range of defined clinical skills *before* and *after* the BSc using a five- point scale that is based on that used by ACCS trainees.  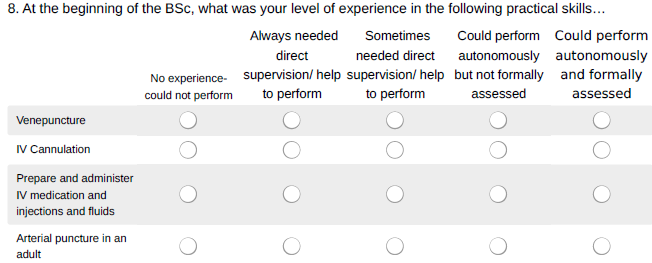  **(B)** Students rate general perceptions of the course using a standard five point agreement scale:  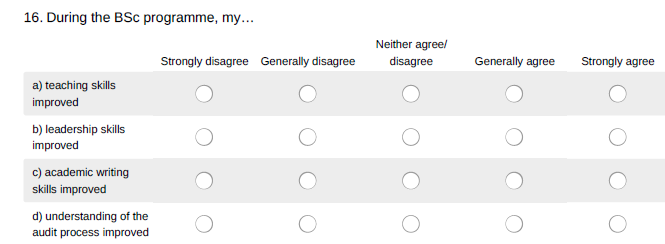  **(C)** Supervisors were requested to rate student competence using a scale to determine equivalence or superiority with various stages of training. This was extended to FY2 level, to reflect anecdotal reports of some students operating to this level in certain domains by the end of their BSc and ED placement. 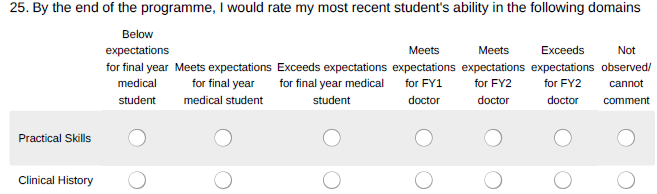 |
| --- |
